# Supplementary material for: Geochemical and Metagenomic Characterization of Jinata Onsen, a Proterozoic-Analog Hot Spring, Reveals Novel Microbial Diversity including Iron-Tolerant Phototrophs and Thermophilic Lithotrophs
Source: Microbes Environ. 2019 Aug 14;34(3):278–92. doi: 10.1264/jsme2.ME19017 (PMC6759342; doi:10.1264/jsme2.ME19017)
Supplement: Supplementary file 1 [file 34_278_s1.pdf]

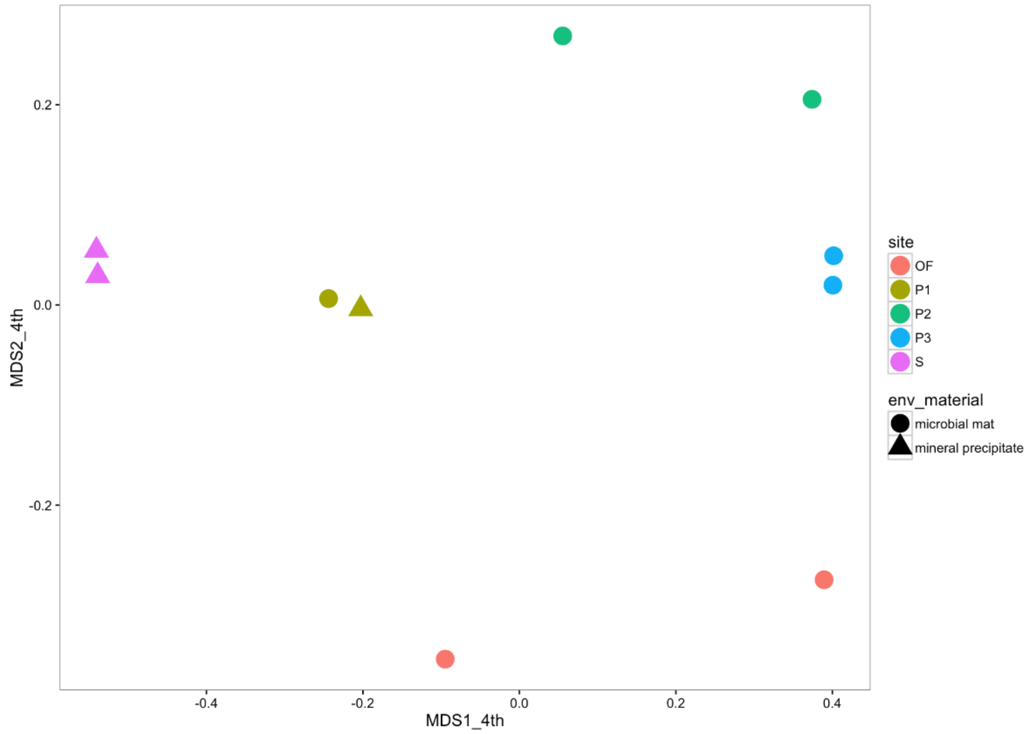

Supplemental Figure S1:
Multidimensional scaling plot of Jinata samples. Each point represents the recovered microbial community from a given sample, with sites identified by color and sample type by shape. Samples plotting close to each other are relatively more similar in community composition. Abundance data are transformed by the 4th root to down-weight the effect of abundant taxa. Stress value is 0.0658.

Supplemental Figure S2:
Microscopy images of sediment (Source Pool and Pool 1) or mat (Pool 2, Pool 3, and Out Flow). Left are light microscopy images. Center and right are fluorescence images. At center, blue signal is DAPI-stained (Excitation: 365nm, Emission: BP445~50nm). At right, red is autofluorescence signal of Cyanobacteria (BP395~440nm, LP470nm). Scale bars 50 µm.

Supplemental Table S1: Geochemistry and brief description at sampling sites along the flow path of Jinata Onsen as discussed in the text.

	pH	T (°C)	Fe(II) (µM)	DO (µM)	DIC (mM)	DOC (mM)	Descriptions
**Source Pool**	**5.4**	**60-63**	**260**	**4.7 (source) 39**	**Not measured**	**Not measured**	**Fluffy red iron oxide precipitate**

				**(surface)**			
**Pool 1**	**5.8**	**59-59.5**	**265**	**58**	**5.51 ± 0.28**	**1.31 ± 0.18**	**Reddish precipitate and streamers in shallower regions, more yellowish deeper**
**Pool 2**	**6.5**	**44.5-54**	**151**	**134**	**2.09 ± 0.11**	**0.76 ± 0.10**	**Iron oxide-coated microbial mats. Orange to orange-green.**
**Pool 3**	**6.7**	**37.3-46**	**100**	**175**	**1.79 ± 0.09**	**0.70 ± 0.10**	**Green or mottled orange-green microbial mats, commonly with 1-5cm finger-like morphology.**
**Outflow**	**6.5**	**27-32**	**45**	**234**	**Not measured**	**Not measured**	**Ocean water within mixing zone at high tide, with constant flow of spring water from Pool 2. Thin green microbial mats.**

Supplemental Table S2: Gas composition of bubbles collected from the Source Pool at Jinata Onsen.

	Average of gas compositions (percent composition)								
Sampling dates (mm/dd/yyyy)	Measurement number	N_2	SE	O_2	SE	CH_4	SE	CO_2	SE

10/03/2017	2	30.5	4.6	0.10	0.01	0.04	0.01	69.3	4.6
04/13/2018	4	55.5	5.5	0.07	0.04	0.05	0.01	44.4	5.0

Supplemental Table S3:
Diversity metrics of Jinata sequencing. Diversity metrics calculated for both 99% and 97% sequence identity cutoffs for assigning OTUs.

**Sample:**	**Reads:**	**OTUs (99%):**	**Good's Coverage (99%):**	**Shannon Index (99%):**	**Inverse Simpson (99%):**	**OTUs (97%):**	**Good's Coverage (97%):**	**Shannon Index (97%):**	**Inverse Simpson (97%):**
**Source Pool A**	48680	18832	0.665	10.443	35.146	6951	0.907	7.996	17.361
**Source Pool B**	18235	7772	0.646	10.388	68.018	4139	0.844	8.651	31.822
**Pool 1 A**	96268	25305	0.788	10.172	53.546	11734	0.920	8.403	28.691
**Pool 1 B**	56672	13835	0.797	8.813	26.975	5598	0.933	6.818	13.827
**Pool 2 A**	35690	12489	0.713	9.625	22.248	7352	0.855	8.271	16.600
**Pool 2 B**	4454	2274	0.560	9.066	49.599	1729	0.689	8.104	27.390
**Pool 3 A**	28273	11334	0.665	10.046	35.705	6766	0.824	8.403	20.034
**Pool 3 B**	2076	1166	0.522	8.832	75.900	786	0.699	7.489	35.312
**Outflow A**	31980	18486	0.497	11.989	64.318	11994	0.712	10.538	34.881
**Outflow B**	32465	10896	0.713	9.281	25.691	5918	0.857	7.133	11.585

Supplemental Table S4:
16S rRNA gene amplicon data as OTU table with sequences.

Supplemental Table S5:
16S rRNA gene amplicon data as relative abundance binned at the class level.

Supplemental Table S6:
1. High- and medium-quality metagenome-assembled genomes (MAGs) (>50% completeness and <10% contamination) recovered from Jinata Onsen. Predicted taxonomy based on placement in reference phylogeny as presented in Figure 4 and by GTDB-Tk (87). Optimal growth temperatures predicted following methods from (Li, G., Rabe, K.S., Nielsen, J. and Engqvist, M.K., 2019. Machine learning applied to predicting microorganism growth temperatures and enzyme catalytic optima. *BioRxiv*, p.522342).

Supplemental Table S7:

Presence of genes involved in aerobic respiration, hydrogen- and iron-oxidation, and carbon fixation in MAGs discussed in the text, with NCBI accession numbers provided.
