# Supplementary figures and images for: Geochemical and Metagenomic Characterization of Jinata Onsen, a Proterozoic-Analog Hot Spring, Reveals Novel Microbial Diversity including Iron-Tolerant Phototrophs and Thermophilic Lithotrophs
Source: Microbes Environ. 2019 Aug 14;34(3):278–92. doi: 10.1264/jsme2.ME19017 (PMC6759342; doi:10.1264/jsme2.ME19017)

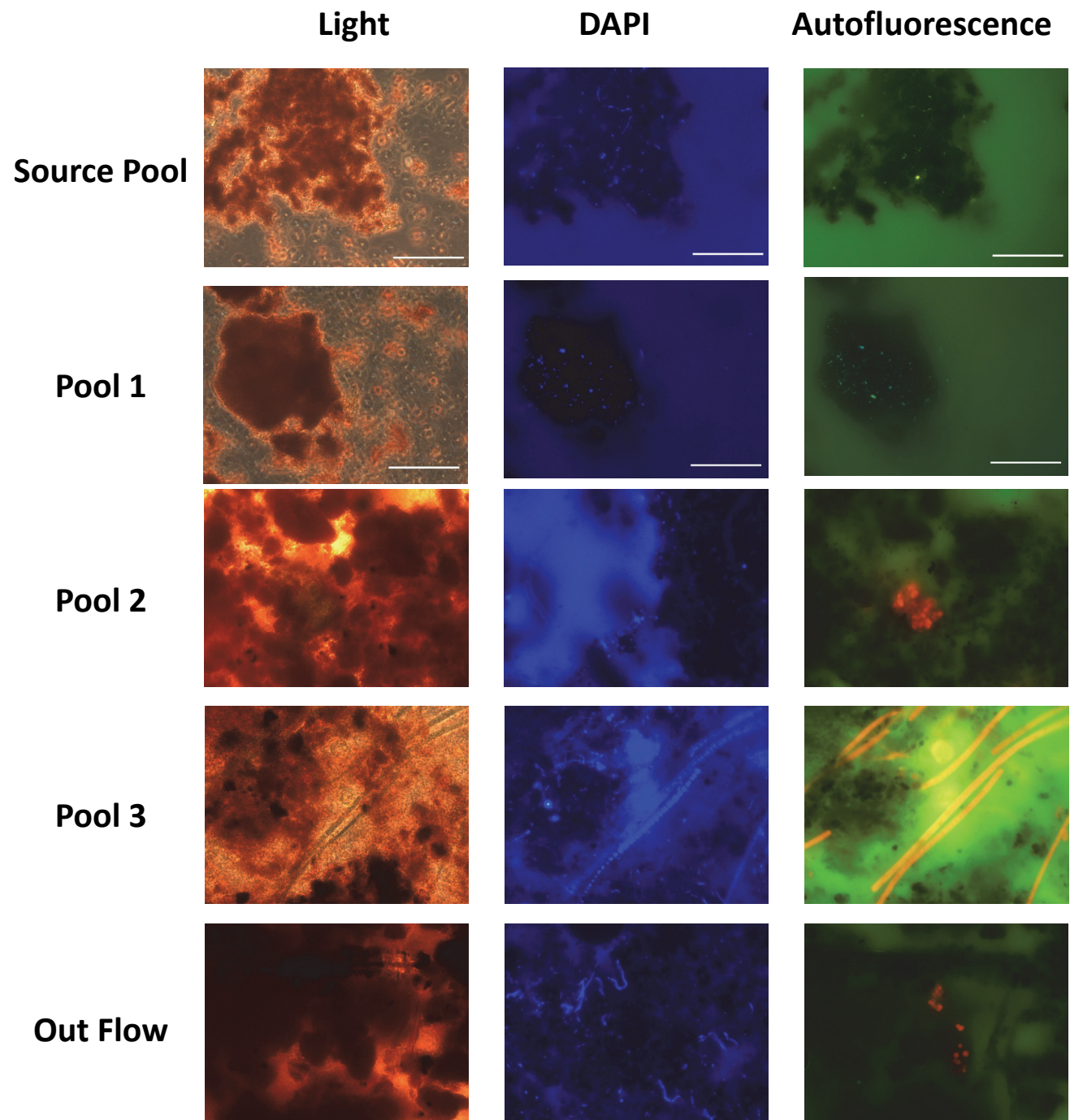

Supplement: Supplementary file 2 [file 34_278_s2.pdf]
